# Global COVID-19 pandemic demands joint interventions for the suppression of future waves

**DOI:** 10.1073/pnas.2012002117

**Published:** 2020-09-28

**Authors:** Ruiyun Li, Bin Chen, Tao Zhang, Zhehao Ren, Yimeng Song, Yixiong Xiao, Lin Hou, Jun Cai, Bo Xu, Miao Li, Karen Kie Yan Chan, Ying Tu, Mu Yang, Jing Yang, Zhaoyang Liu, Chong Shen, Che Wang, Lei Xu, Qiyong Liu, Shuming Bao, Jianqin Zhang, Yuhai Bi, Yuqi Bai, Ke Deng, Wusheng Zhang, Wenyu Huang, Jason D. Whittington, Nils Chr. Stenseth, Dabo Guan, Peng Gong, Bing Xu

**Affiliations:** ^a^Ministry of Education Key Laboratory for Earth System Modeling, Department of Earth System Science, Tsinghua University, 100084 Beijing, China;; ^b^Tsinghua Urban Institute, Tsinghua University, 100084 Beijing, China;; ^c^Center for Healthy Cities, Institute for China Sustainable Urbanization, Tsinghua University, 100084 Beijing, China;; ^d^MRC Centre for Global Infectious Disease Analysis, Department of Infectious Disease Epidemiology, School of Public Health, Faculty of Medicine, Imperial College London, W2 1PG London, United Kingdom;; ^e^Department of Land, Air, and Water Resources, University of California, Davis, CA 95616;; ^f^Department of Urban Planning and Design, The University of Hong Kong, Hong Kong, 999077;; ^g^Center for Statistical Science, Tsinghua University, 100084 Beijing, China;; ^h^Department of Industrial Engineering, Tsinghua University, 100084 Beijing, China;; ^i^CAS Key Laboratory of Pathogenic Microbiology and Immunology, Institute of Microbiology, Chinese Academy of Sciences, Beijing 100101, China;; ^j^CAS Center for Influenza Research and Early-warning (CASCIRE), Chinese Academy of Sciences, Beijing 100101, China;; ^k^CAS-TWAS Center of Excellence for Emerging Infectious Diseases (CEEID), Chinese Academy of Sciences, Beijing 100101, China;; ^l^State Key Laboratory of Infectious Disease Prevention and Control, Collaborative Innovation Center for Diagnosis and Treatment of Infectious Diseases, National Institute for Communicable Disease Control and Prevention, Chinese Center for Disease Control and Prevention, 102206 Beijing, China;; ^m^China Data Institute, Ann Arbor, MI 48108;; ^n^School of Geomatics and Urban Spatial Informatics, Beijing University of Civil Engineering and Architecture, 102616 Beijing, China;; ^o^Institute of High Performance Computing, Department of Computer Science and Technology, Tsinghua University, 100084 Beijing, China;; ^p^Centre for Ecological and Evolutionary Synthesis, Department of Biosciences, University of Oslo, N-0316 Oslo, Norway

**Keywords:** climate, human behavior, disease transmission, hierarchical intervention network, international collaboration

## Abstract

Emerging evidence suggests a resurgence of COVID-19 in the coming years. It is thus critical to optimize emergency response planning from a broad, integrated perspective. We developed a mathematical model incorporating climate-driven variation in community transmissions and movement-modulated spatial diffusions of COVID-19 into various intervention scenarios. We find that an intensive 8-wk intervention targeting the reduction of local transmissibility and international travel is efficient and effective. Practically, we suggest a tiered implementation of this strategy where interventions are first implemented at locations in what we call the Global Intervention Hub, followed by timely interventions in secondary high-risk locations. We argue that thinking globally, categorizing locations in a hub-and-spoke intervention network, and acting locally, applying interventions at high-risk areas, is a functional strategy to avert the tremendous burden that would otherwise be placed on public health and society.

## Significance

By linking seasonality of climate and changing human behavior, we demonstrate that collaboration on global efforts for prompt and intensive intervention is fundamental to coping with future pandemic waves of COVID-19. We propose that this collaboration can be started in locations with typically high population density and international travel, followed by other high-risk locations. We believe this tiered intervention strategy can greatly integrate global efforts and is effective and practical to improve the global emergency response to COVID-19 and many other infectious diseases.

COVID-19 has now become a global pandemic (1). Outside of China, where the first cases were reported, countries experiencing a high risk of infection include the United States, Brazil, and Russia (2). Given the multiple epicenters of the pandemic and mounting toll of the epidemics, it is imperative to seek optimal interventions from a broader, more integrated perspective.

Many countries have taken multisectoral approaches to mitigate the rapid transmission of COVID-19. Among them, social distancing and lockdown suppression appear to be the most effective actions (3–7). However, the intensity and implementation time of these actions varied considerably across countries in the initial wave, which may explain much of the variation in infection rates that exists between populations and countries. However, a pandemic is defined by its global spread, and the effectiveness of coordinating global suppression efforts in subsequent waves of the pandemic has not yet been fully projected.

Prior work has implicated the role of community transmission and case importation in modulating transmission dynamics of the virus (8, 9). In light of this, we argue that transmission dynamics in the following epidemics will be largely dependent on the seasonality of climate and changing human behavior such as social contacts and movement. The risk of community transmission increases with the fraction of social contacts that are inside the local community (e.g., households, workplace), which, in turn, can be modulated by seasonal climate conditions. Furthermore, population movements facilitate the spatial dissemination of the disease, allowing earlier case importation and onset of local transmission. Thus, the current challenge is to link these mechanisms in order to improve projections of the overall transmission dynamics and inform decisions on the optimal implementation of intervention strategies with a global perspective.

In this study, we propose a hierarchical intervention strategy for collaborative global suppression efforts. We have developed a mathematical model that projects the transmission dynamics of COVID-19 among 59 high-risk locations in the subsequent pandemic seasons. We explicitly incorporated two mechanisms that are likely to shape the postpandemic trajectory of COVID-19: 1) the seasonality of local climate conditions, through their impact on changing social behavior in terms of staying indoors, which dominates the community transmission risk over time and locations, and 2) international travel, which facilitates the spatial diffusion, and hence the risk, of case importation. To evaluate the gains that could be achieved through these joint interventions, we applied the model to a variety of scenarios with differing strategies, intensities, and durations of intervention on community transmission and international travel.

There are now many modeling analyses which focus on optimizing interventions to control outbreaks of COVID-19 (5, 10–12), but few efforts have been made to test an effective framework of global coordination to contain pandemic outbreaks. Unlike local implementation of interventions and health care efforts, international travel and climate, which are known to influence infectious disease transmission, can be clearly evaluated at the global scale. Concerns are growing over the negative impacts of sustained social lockdowns and travel restrictions on economies (13), so it is important to explore control approaches that are both rapid and effective. Here, we present an approach involving a highly effective 8-wk global coordinated and intensive approach targeted at specific major international hubs that may more effectively control pandemic outbreaks.

## Results

**Fig. 1.**
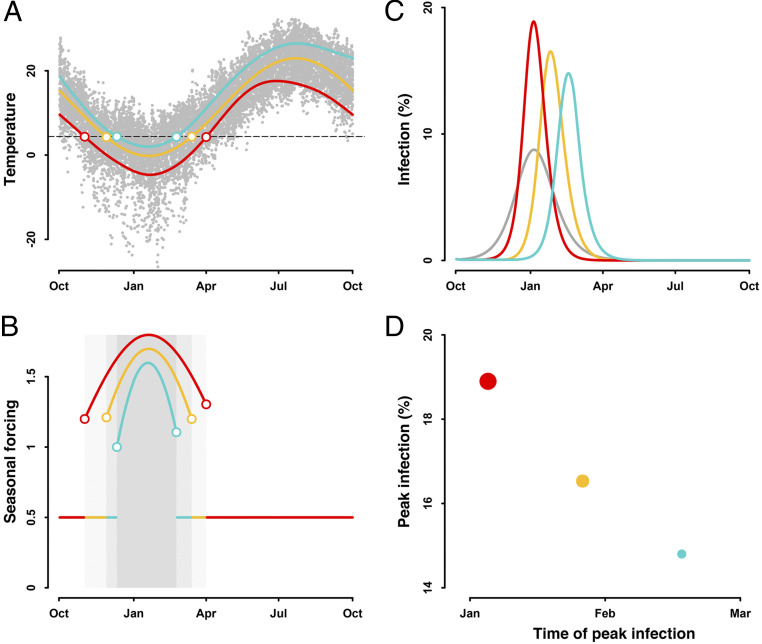
Effect of seasonal climate conditions on trajectory of infection. (*A*) Wintertime varies across locations, that is, the longer (red) or shorter winter (green) compared with the overall duration of the winter season (yellow). Colored circles indicate the start and end of the winter season. Gray dots show the daily temperature. Dashed line is the temperature threshold for winter. (*B*) The cold weather in winter, through driving people indoors for a longer time, inflates the forcing on transmission. Shaded areas mark the winter seasons. Seasonal forcing suppresses the transmission by 50% and is invariant in other seasons. (*C*) This climate-modulated transmission risk modulates the spread of infection. Gray line shows the trajectory of infection resulting from a seasonally invariant risk of transmission, assuming $R_{0}\;=\;2$. The Susceptible–Exposed–Infectious–Recovered (SEIR) model is initialized with *S*(0) = 0.999, *E*(0) = 0.001, *I*(0) = 0, and *R*(0) = 0. Trajectories of infection are simulated using values for 1/δ = 5.2 d, 1/γ = 7 d and $R_{0}\;=\;0$. Effect of seasonal forcing on transmission is modeled through $R\left(t\right)=R_{0}\theta \left(t\right)$, where θ(t) and R(t) is the seasonal forcing and transmissibility on day *t*, respectively. (*D*) Time and magnitude of peak infections vary across locations. Peak infection is delayed and lowered in locations with a shorter duration of winter season. Circle size shows the magnitude of peak infection.

The transmission risk and spread of infections can be modulated by local climate conditions. Wintertime in temperate and subtropical climates typically falls between October and April (Fig. 1*A*). In response to the cold weather, people spend more time indoors during winter. This behavior inflates the risk of transmission (Fig. 1*B*) and spread of infection (Fig. 1*C*). This effect of seasonal climate conditions, through changing human behavior, on transmission of diseases varies across locations. The peak infection is delayed and lowered in locations with a shorter and warmer winter (Fig. 1*D*). More specifically, a longer and colder winter (Fig. 1*A*, red line) increases the time indoors, leading to a persistent inflation of transmission (Fig. 1*B*, red line) and a larger and earlier peak of infection (Fig. 1*C*, red line). By contrast, a shorter and warmer winter (Fig. 1*A*, green line) reduces the time indoors, and thereby lowers the transmissibility (Fig. 1*B*, green line), delaying and decreasing the peak infection (Fig. 1*C*, green line).

**Fig. 2.**
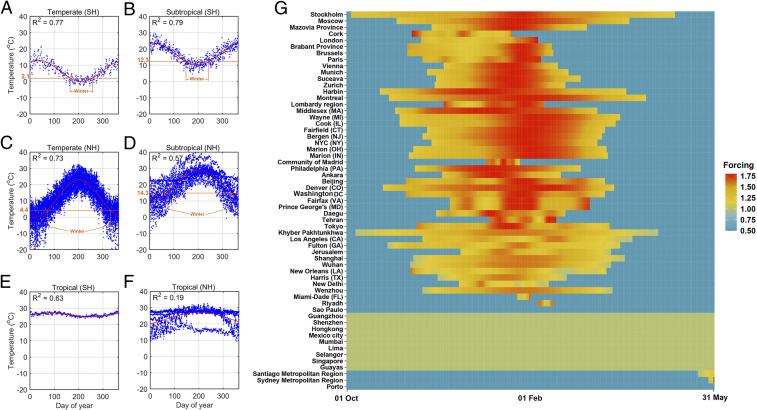
Best fit of daily temperature and seasonal forcing on community transmission. (*A*−*F*) Best fit of daily temperature in 2019 among locations in temperate, subtropical, and tropical climates in Northern (NH) and Southern (SH) Hemispheres. Wintertime in the temperate and subtropical climates is marked. (*G*) Locations are ordered by latitude. Magnitude of seasonal forcing is distinguished by color.

Seasonal forcing on community transmission of COVID-19 in a wide-ranging set of locations in temperate and subtropical regions is shown in Fig. 2 *A*–*D*. Aligned with the above mechanism, variation of seasonal forcing on community transmission is characterized by an inflation of the transmission activity in the wintertime and a suppression in other seasons (Fig. 2*G*). The intensity, initial time, and duration of this inflation period, however, varies across locations. Locations in temperate climates tend to have greater inflation intensity, coupled with an advancing and extending duration associated with the latitude. This spatial variation is suggestive of the earlier and longer indoor stay in high-latitude locations. By contrast, Fig. 2 *E* and *F* shows an insignificant seasonal fluctuation of temperature in tropical locations, and thereby no seasonal fluctuation of transmission activity.

Additionally, the airline passenger flow is positively correlated with the arrival time of the epidemic (*SI Appendix*, Fig. S1*A*). More specifically, locations with more international trips are more likely to have an earlier onset of local transmission, suggesting travels increase the risk of case importation. This variation in passenger flow, coupled with that in population size (*SI Appendix*, Table S1), allows us to rank locations into two-tiered risk categories. The 15 hub locations with higher population densities and airline trips than the median of all locations, which exhibit greater responsiveness to epidemic onset, are collectively designated as the Global Intervention Hub (GIH). The remaining 44 locations with comparatively lower densities and fewer airline trips were assigned as secondary high-risk locations (*SI Appendix*, Table S1).

**Fig. 3.**
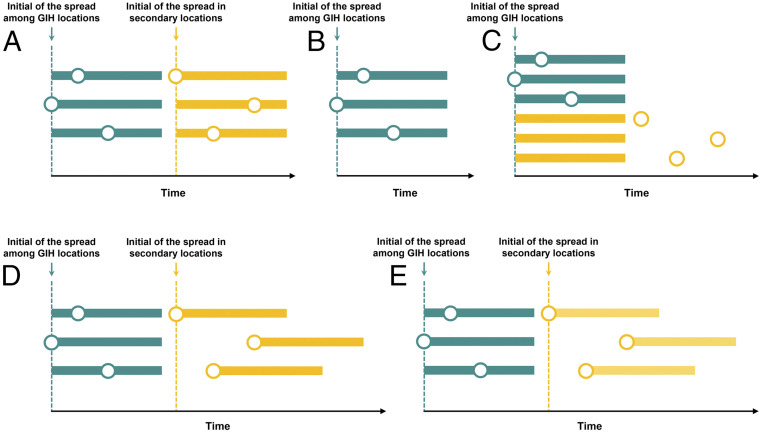
Scenario sets of intervention. We applied five intervention strategies: (*A*) the prompt and joint intervention among GIH locations, followed by joint intervention in secondary locations, (*B*) the prompt and joint intervention implemented only among GIH locations, (*C*) interventions initialized simultaneously among all locations, (*D*) the prompt and joint intervention among the GIH locations, followed by interventions in secondary locations which initialized according to location transmission risk, and (*E*) the intensive intervention in the GIH locations followed by a moderate intervention in secondary locations. Color distinguishes the intervention implemented among GIH (green) and secondary (yellow) locations. Circles and horizontal bars indicate the initial time of transmission and duration of intervention in each location. Dashed lines show the tiggering time of intervention.

**Fig. 4.**
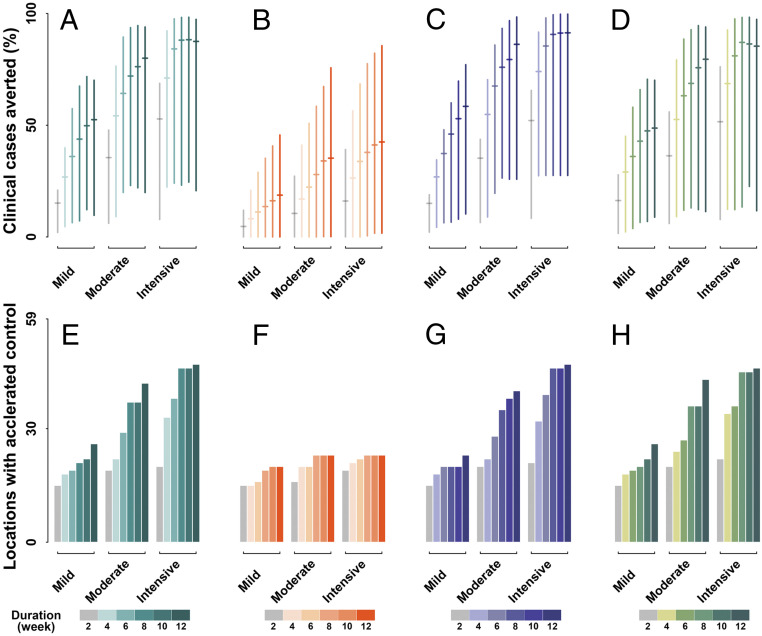
Impact of interventions. Interventions are implemented through three scenario sets: (*A* and *E*) sequentially between GIH and secondary locations, (*B* and *F*) only in GIH locations, (*C* and *G*) simultaneously among all locations, and (*D* and *H*) sequentially between GIH and secondary, but initialized independently in secondary, locations. For each scenario set, mild, moderate, and intensive intervention reduces community transmission and international travel by 20%, 50%, and 80%, respectively. Intervention durations of 2 wk to 12 wk are considered, in increments of 2 wk. Effectiveness is evaluated in terms of the proportion of clinical cases averted and number of locations where the goal of effectively reducing incidence to <10 cases per day could be achieved in advance as compared with projections in the absence of interventions.

Consistent with this, we propose a hub-and-spoke organization of interventions that applies a lead intervention first at all GIH locations, followed by interventions later at all secondary locations (set 1) (Fig. 3*A*). Transmissions within a first location signal the onset of implementation of interventions, which are applied jointly at all locations within the category, GIH or secondary. With a 2-wk mild intervention that reduces community transmission and international travel by 20%, the model projected that a median of 15.11% (90% CI: 2.02 to 20.92%) of clinical cases would be averted (Fig. 4*A*), coupled with the accelerated reduction of incidence to <10 cases (taken as “the effective control”) in 15 locations (Fig. 4*E*). Sustaining and intensifying this intervention strategy is associated with a substantial alteration to the pandemic trajectory. Extending the mild intervention to 12 wk would avert a median of 52.44% (9.69 to 70.12%) cases and lead to effective control of transmission in 26 locations. Lifting to a moderate intensity for 12 wk would lead to a median of 79.95% (19.89 to 93.94%) cases averted, accelerating the process of effective control in 42 locations. It is worth noting that an 8-wk intensive intervention in the initial period among the GIH locations and a subsequent 8-wk intensive intervention among the secondary locations would be the most promising strategy. Our model indicates that this strategy would result in a median of 88.02% (23.18 to 98.25%) fewer cases, with advanced control of transmission reaching across 46 locations. Unlike when all locations are treated together, implementing intensive intervention for a longer duration would not make any apparent contribution to improving this effectiveness.

Collaboration among GIH and secondary locations would be necessary. In the absence of intervention among secondary locations (set 2) (Fig. 3*B*), we projected a median of 42.45% (1.70 to 85.59%) averted infections (Fig. 4*B*) and 26 locations with effective control of transmission in advance (Fig. 4*F*) through a 12-wk intensive intervention. Additionally, the simultaneous initialization of interventions across all high-risk locations (set 3) (Fig. 3*C*) would make a marginal contribution to improving the effectiveness of intervention. Assuming an 8-wk intensive intervention, our projections suggest an increase of 2.58% in averted infections (Fig. 4*C*) and no additional locations with advanced effective control (Fig. 4*G*), compared with the optimal strategy.

Note that population size and international travel among secondary locations are expected to be more diverse and complex than for those in GIH, indicating that initializing interventions simultaneously among them is less practical. Given this, we investigated a more flexible intervention strategy, allowing secondary locations to tailor the triggering time of local interventions (set 4) (Fig. 3*D*). Our findings show that this strategy would also produce a considerable reduction in the disease burden spanning multiple locations (Fig. 4 *D*–*H*). With an 8-wk intensive intervention, we projected a median of 87.02% (13.41 to 98.23%) averted cases with accelerated effective control in 45 locations. This effect is equivalent to that of joint intervention being initialized simultaneously among secondary locations. This finding indicates that prompt and joint interventions among the GIH in the initial period would allow for greater flexibility in the secondary locations tailoring the triggering time of intervention. However, this flexibility does not mean that intervention intensity across secondary locations could be reduced (set 5) (Fig. 3*E*). Softening the strategy to an 8-wk moderate intervention would lead to a drop of ∼10% in infections averted and six fewer locations with advanced effective control of transmission (*SI Appendix*, Fig. S2), compared with an intensive intervention.

## Discussion

Applying interventions at high-risk areas through a hub-and-spoke network is the key step to improving our emergency response and coping with future waves of the COVID-19 pandemic. Although the intensity and duration of intervention is highly dependent on the socioeconomic status of each location, our findings suggest that global pandemics are far from uncontrollable with global collaboration. To facilitate this, locations in the GIH should take the lead on mitigating community transmission and international travel. Among these GIH locations, outbreaks occurring in any single location would be an early warning signal that informs the decision for a prompt response in the remaining locations.

Our projections imply that considerable gains could be achieved by implementing an 8-wk intensive intervention in the GIH followed by an identical intervention among other high-risk locations. Primarily, this aggressive intervention strategy would reduce the overall disease burden by ∼90%, advancing the goal of effective control across 46 locations. Practically, the initial and joint intervention within the GIH would offer more flexibility to the following mitigation in secondary high-risk locations. Once the GIH locations had collaborated on their efforts in the very early stage of the pandemic, it would be possible for the remaining high-risk locations to tailor the triggering time of mitigation according to the local transmission risk. Whenever mitigation is implemented, it should be intensive. The superior performance of this intervention relay is supported by the comparable performance of simultaneous intervention among all locations.

In light of these results, we must emphasize that within-community transmission and travel-related risk of spreading the disease are highly likely to emerge in every country. The strategic collaboration on the global effort is therefore imperative. Indeed, travel from Wuhan is strongly associated with the unprecedented diffusion of the disease to other Chinese cities (14–16). However, this nationwide spatial transmission mechanism does not, thus far, appear to be the primary driver shaping the global transmission dynamics. This is largely in agreement with the mounting evidence of early community spreading prior to the official reports of the first cases in many countries (17). It is notable that recent studies appear to hypothesize that China was the epicenter seeding the outbreak elsewhere across the world (18–20), possibly mirroring the knowledge gap in the transmission dynamics on the global scale.

We recommend a strategic two-tiered collaborative intervention framework aimed at achieving a prompt and effective reduction of transmissions in the early stage of the global pandemic. Our projections document the comprehensive effectiveness of this strategy, but also great variation among locations. This variation does not count against our strategic recommendation, but indicates the necessity of tailoring a location-specific intervention strategy to further reduce the local transmission. Alongside the two-tiered collaborative strategy, involving location-specific actions would form a multitiered intervention paradigm, leveraging global and local contribution to the reduction of disease burden.

Our study is subject to several limitations. First, the most effective action could vary across locations, age, and gender (10, 21, 22). In some high-risk locations, combined actions would be optimal (4, 12). To help tailor and optimize the intervention actions in specific settings, further work should provide scientific evidence on the effectiveness of each action. Second, alternative modes of indoor transmission, such as those happening in hospitals, have not been explicitly considered. By using the household transmissibility, we projected a baseline transmission risk over the community. However, this community transmission risk could have been underestimated in the absence of context-specific characterization of other possible modes. Third, aerosol transmissions (23), population susceptibility (24), immunity (25), and asymptomatic infections (26) are potentially fundamental modulators in the endemic stage of the disease. Their impact on the COVID-19 trajectory is inconclusive but warrants further studies and should be considered in long-term projections. Finally, we proposed a dominance of temperature on the duration of indoor stay, leading to a seasonal transmission risk. Aligned with this, we explicitly modeled the temperature-dependent dynamics of risk in the wintertime, while we assumed a 50% reduction in other seasons. It is possible to observe a moderate transmission peak in the summer for some specific settings such as documented in seasonal flu (27). Nevertheless, the winter transmission peak is typical in temperate and subtropical climate, while the secondary peak occurred occasionally in summer in only some settings. The potential mechanism underlying the summer peak is out of the scope of this study. Taken together, the extent to which we can successfully mitigate the next waves of the pandemic is strongly dependent on how all nations collaborate. Importantly, the disease burden of COVID-19 could be significantly reduced through the initial intervention, as coordinated by the GIH in the early stages. This intervention effect would be consolidated by timely and intensive actions among remaining high-risk locations.

## Materials and Methods

### Global High-Risk Locations and Intervention Hub.

High-risk locations were assumed to dominate the overall transmission dynamics of COVID-19 from the global perspective. To select these locations, we used the number of cumulative cases as the primary criteria and took the following steps. Initially, we selected high-risk countries with >10,000 cumulative cases as of April 24, 2020. For completeness, countries with distinct climates and thereby seasonality of transmission, such as Australia and Malaysia, were additionally included. There were a total of 2,485,256 confirmed cases among the selected countries, accounting for 92.75% of the cases worldwide.

Next, we identified locations with the highest numbers of cumulative cases within each selected country (hereafter “high-risk locations”), except for the United States, China, and India, where multiple locations were included. Specifically, given the excess cases in the United States, we used 10,000 as the threshold to identify high-risk states. Within each of them, locations with the highest number of cumulative cases were designated as high-risk locations. Furthermore, seven Chinese cities expected to have a high level of risk (18), that is, Wuhan, Beijing, Shanghai, Guangzhou, Shenzhen, Wenzhou, and Harbin, were included. Additionally, Mumbai and New Delhi have more cumulative infections than other Indian cities; therefore both were included.

A total of 59 locations were analyzed in our study. These locations are, depending primarily on the country-specific reporting system of COVID-19 cases, cities (39 out of 59 locations), counties (10/59), provinces (4/59), regions (3/59), states (1/59), communities (1/59), and countries (1/59). Details of the 59 locations analyzed are provided in *SI Appendix*, Table S1.

We designed a hub-and-spoke network, assigning these high-risk locations to either GIHs or secondary high-risk locations according to their population density and airline passenger flows in 2019. The GIH locations, with higher density and flows than the median among all locations, should take the lead on prompt and joint intervention (see *Model Simulations*).

### Climate-Driven Variation in Staying Indoors.

Climate is a crucial modulator of human behavior such as movement and social contact. It is worth noting that we do not explicitly model the climate dependency of pathogen transmission, as the climate−transmission interaction is still inconclusive (28, 29). Instead, we proposed that seasonality of temperature would drive people’s behavior of staying indoors and the probability of being infected indoors. For example, in some climates, people spend more time indoors and hence are more likely to get infected when indoors during winter. Therefore, the dynamics of the time that people spend indoors and the probability of indoor infection align with those of the local temperature.

To characterize the seasonality of temperature, we obtained daily temperature data for all 59 locations from ERA5 (fifth generation of European Centre for Medium-Range Weather Forecasts [ECMWF] atmospheric ReAnalysis) Daily aggregates of climate reanalysis data (30). Depending on the hemisphere and climate, the locations were assigned to one of six zones, that is, temperate, subtropical, and tropical climates in the Southern and Northern Hemispheres. In each zone, we fitted the temperature data over time across locations using Fourier functions [**1**] to characterize the general seasonality.

$$temp_{i}\left(t\right)\;=\;a_{0,i}\;+\;a_{1,i}\;\cos\left(w_{i}t\right)\;+\;b_{1,i}\;\sin\;\left(w_{i}t\right),$$ [1]

where $temp_{i}$ is the temperature in locations in zone i, and $a_{0,i},\;a_{1,i},\;b_{1,i},\;\text{and}\;w_{i}$ are model parameters. The best-fit model parameters are listed in *SI Appendix*, Table S2.

Fig. 2 *A*–*D* shows the typical seasonality of temperature in temperate and subtropical climates. We assumed that the dynamics of the time spent indoors would follow this seasonality in winter. That is, we would expect an increasing duration of time indoors during wintertime as it gradually becomes colder than autumn. We assumed a 60 to 90% and 50 to 80% fluctuation of time spent indoors during winter in temperate and subtropical climates, respectively. With the dynamics of daily temperature as a weighting factor, we interpolated the duration of time spent indoors on each day $\left(T_{t}\right)$. This temperature-driven variation in time spent indoors was translated to the probability of being infected when indoors according to

$$P\left(T_{t}\right)\;=\;1-{\left(1-p\right)}^{T_{t}/T_{0}}.$$ [2]

The probability of inhaling the virus per breath, p, is calculated as $p\;=\;e^{-BK}$, where the constant *B* = 0.99 and the extent to which virus-laden air exhaled by the patient is diluted is *K* = 10. *T*_0_ is the time per breath (0.75 s), and $T_{t}$ is the duration of time spent indoors on day t. Assuming a 12-h indoor stay, we projected the baseline estimate of the probability. In this way, seasonal forcing on community transmission (see below) during winter was defined as the inflated probability of being infected on day t as compared with the baseline estimate.

### Community Transmission Risk.

Community transmission risk was captured by the within-household transmissibility, $R_{e}^{hh}$, multiplied by a seasonal forcing, θ(t), that is, $R\left(t\right)\;=\;R_{e}^{hh}\theta \left(t\right)$. Here, we assumed that within-household transmission would be the primary mode of community transmission in the following pandemic waves. The logic of this assumption is consistent with the mounting evidence indicating the within-household transmission/dynamics (31, 32).

The maximum effective reproductive number in a partially susceptible population, $R_{e}^{hh}$, depends on the household structure in each country. We collected the family structure, household size, and composition from United Nations records (33). We assumed a six-member household and estimated $R_{e}^{hh}$ according to

$$R_{e}^{hh}\;=\;\frac{H_{1}}{H}\times 0+\frac{H_{2}}{H}\times 0.5+\frac{H_{3}}{H}\times 1+\frac{H_{4}}{H}\times 1.5+\frac{H_{5}}{H}\times 2\frac{H_{6}}{H}\times 2.5,$$ [3]

where H is the total number of households, estimated by$H\;=\;\sum_{i=1}^{hh_{max}}H_{i}$, $H_{i}$ is the number of households with i members, and $hh_{max}$ is the maximum number of members in the household. By assuming a homogeneous household structure across locations within a country, we used this country-specific estimate as a proxy for $R_{e}^{hh}$ in the corresponding high-risk locations.

The function θ(t) reflects the seasonal forcing on community transmission risk. The risk increases with the fraction of social contacts that are inside the local community, such as households. Therefore, the temporal dynamics of θ(t) follow the seasonality of temperature during winter in temperate and subtropical climates, leading to an inflated seasonal forcing on transmission (see above) as people are spending more hours indoors with poor ventilation. Note that spring, summer, and autumn in temperate and subtropical climates are periods of low transmission activity, assuming a resemblance to the seasonality of flu. Therefore, we did not explicitly project the temperature-driven variation in the duration of time spent indoors to the seasonal forcing on transmission. Instead, we assumed a constant seasonal forcing that suppressed transmission by 50%. Additionally, Fig. 2 *E* and *F* indicates an insignificant seasonal fluctuation of temperature in tropical locations. Consistent with this, we assumed no seasonal fluctuation of transmission activity, and thereby assigned a unit seasonal forcing over the pandemic season. The dynamics of seasonal forcing across locations are presented in Fig. 2*G*.

### International Travel.

To simulate the spatiotemporal dynamics of the disease on a global scale, we extracted monthly airline transportation data between all high-risk locations in 2019 from the International Air Transport Association, which has 100% coverage of global airline market. Here, we assumed that international airline transportation would progressively return to the routine operating levels that existed prior to the initial pandemic, that is, in the absence of travel restrictions. We therefore used the 2019 data as a proxy for the travel dynamics in subsequent pandemic years. In the model simulations, this monthly dataset was interpolated using spline functions to reflect its daily fluctuations as follows. First, we transformed the monthly dataset to cumulative monthly transportation data, which increases over time in 1-mo increments. Next, we interpolated this cumulative monthly data by spline functions and then discretized the spline with the time step of 1 d to extract the cumulative daily airline transportation. Finally, we took successive differences of this cumulative daily data to get the daily airline transportation.

With this passenger flow data, we evaluated the association between travels and the arrival time of local transmission. The arrival time is defined as the first day 10 cases are recorded in the initial wave, reflecting the onset of local transmission. Accordingly, the travel−arrival association, fitted by spline function, implicates the role of international travels in shifting the onset time of local transmission.

### Model Simulations.

The metapopulation model incorporates seasonal variations in community transmission and spatiotemporal diffusion across 59 global high-risk locations in the following framework:

$$\frac{dS_{i}}{dt}\;=\;-\frac{\beta S_{i}I_{i}}{N_{i}}\;+\;\sum_{j}\frac{M_{ji}S_{j}}{N_{j}-I_{j}}-\underset{j}{\sum }\frac{M_{ij}S_{i}}{N_{i}-I_{i}}$$

$$\frac{dE_{i}}{dt}\;=\;\frac{\beta S_{i}I_{i}}{N_{i}}-\delta E_{i}+\underset{j}{\sum }\frac{M_{ji}E_{j}}{N_{j}-I_{j}}-\underset{j}{\sum }\frac{M_{ij}E_{i}}{N_{i}-I_{i}}$$

$$\frac{dI_{i}}{dt}\;=\;\delta E_{i}-\gamma I_{i}$$

$$\frac{dR_{i}}{dt}\;=\;\gamma I_{i}$$

$$N_{i}\;=\;N_{i}+\underset{j}{\sum }M_{ji}-\underset{j}{\sum }M_{ij},$$

where $S_{i}$, $E_{i}$, $I_{i}$, $R_{i}$, and $N_{i}$ are the susceptible, exposed, infected, removed, and total populations, respectively, in city *i*. The average incubation period, 1/*δ*, is assumed to be 5.2 d, and the average duration of infection, 1/*γ*, is set to 7 d (5, 34, 35). The rate of transmission, β, is related to the community transmission risk R(t) through β(t) = R(t)/γ. Assuming that infected individuals are quarantined, the spatial diffusion of the disease is represented by the daily number of people traveling from city *i* to *j* $\left(M_{ij}\right)$ in all groups except $I_{i}$. Simulations are initialized with 0.01% of population localized being infected.

We initially applied our model to the baseline scenario, in which there is an absence of any interventions. This means that the overall transmission dynamics are modulated via social contacts within communities and international travel.

Next, we applied three intervention scenarios mitigating community transmission and international travel (Fig. 3 *A*–*C*). To facilitate the analysis, we designed a hub-and-spoke intervention network consisting of the GIH or secondary high-risk locations according to their similarity in terms of population density and airline flows. The hub locations, collectively designated as the GIH, typically have higher population density and greater flows of people, and are therefore much more likely to have larger and earlier outbreaks. Comparatively, the lower density or smaller volume of travel to/from secondary locations is suggestive of a smaller and later outbreak. Consistent with this, we proposed a prompt and joint intervention initiated across hub locations followed by interventions in secondary locations (set 1). That is, locations in the GIH would take the lead on intervention in the first period; other high-risk locations would initiate interventions later in the second period. In each period, we proposed a prompt and joint intervention among locations. This means that, once cumulative infections reached twice the number of initial cases in any location, an intervention would be initiated in the remaining locations within the same tier. By doing so, typical transmission within the first location signals the implementation of intervention in the remaining locations. For simplicity, we assumed the same intervention intensity and duration between the first and secondary periods. That is, interventions in the GIH and secondary locations differentiated only in the time at which they were implemented. To validate set 1, we investigated two other scenarios where prompt and joint intervention was implemented only among GIH locations (set 2) or simultaneously among all locations (set 3).

Across these scenario sets, we examined the impact of various intensities and durations of intervention through the definition of mild, moderate, and intensive interventions, which respectively translate to a reduction of 20%, 50%, and 80% in community transmission risk and international travel. Coupled with each intensity, the duration of intervention was extended from 2 wk to 12 wk in increments of 2 wk.

We applied the proposed model and projected the incidence over the pandemic season, that is, October through May, in the following waves. We jointly estimated the overall number of clinical cases and days to achieve the goal of effective control by reducing the incidence to <10 cases per day. The effect of intervention was evaluated as the proportion of clinical cases averted and the number of locations that achieved effective control in advance, as compared with the baseline scenario.

### Alternative Scenario Sets of Intervention.

Note that the population density and international travel among secondary high-risk locations are expected to be more diverse and complex than for the GIH locations. Secondary locations are therefore less likely to initialize interventions simultaneously. Alternatively, we investigated a more flexible intervention strategy, allowing secondary locations to initialize interventions according to the time when local cumulative cases became twice the number of initial cases (set 4) (Fig. 3*D*). The proportion of cases averted and the locations achieving accelerated control of transmission were examined for a range of intervention intensities and durations.

Additionally, we assumed the same level of intervention intensity between GIH and secondary locations in set 1. To examine the effects of reducing the intensity in the secondary period, we employed a scenario with intensive intervention among GIH locations, but a moderate intervention among the secondary locations (set 5) (Fig. 3*E*). The proportion of cases averted and the locations with accelerated control were examined for various durations of intervention.

## Supplementary Material

Supplementary File

## Data Availability

The data are available upon request from the authors.
