# Supplementary material for: Global COVID-19 pandemic demands joint interventions for the suppression of future waves
Source: Proc Natl Acad Sci U S A. 2020 Sep 28;117(42):26151–7. doi: 10.1073/pnas.2012002117 (PMC7585010; doi:10.1073/pnas.2012002117)
Supplement: Supplementary File [file pnas.2012002117.sapp.pdf]

PNAS
www.pnas.org

Supplementary Information for

Title: Global COVID-19 pandemic demands joint interventions to the suppression of future waves

Ruiyun Li[a,b,c,d,1], Bin Chen[e,1], Tao Zhang[a,b,c], Zhehao Ren[a], Yimeng Song[f], Yixiong Xiao[a,b,c], Lin Hou[g,h], Jun Cai[a], Bo Xu[a,b,c], Miao Li[a], Karen Kie Yan Chan[a], Ying Tu[a], Mu Yang[a], Jing Yang[i], Zhaoyang Liu[g,h], Chong Shen[g,h], Che Wang[g,h], Lei Xu[a,b,c], Qiyong Liu[j], Shuming Bao[k], Jianqin Zhang[l], Yuhai Bi[i], Yuqi Bai[a,b,c], Ke Deng[g,h], Wusheng Zhang[m], Wenyu Huang[a], Jason D. Whittington[n], Nils Chr. Stenseth[a,n,2], Dabo Guan[a,b,c,2], Peng Gong[a,b,c,2], Bing Xu[a,b,c,2]

Corresponding to: Nils Chr. Stensethr: n.c.stenseth@ibv.uio.no; Dabo Guan: guandabo@tsinghua.edu.cn; Peng Gong: penggong@tsinghua.edu.cn; Bing Xu: bingxu@tsinghua.edu.cn.

This PDF file includes:

Supplementary text
Figures S1 to S2
Tables S1 to S2

www.pnas.org/cgi/doi/10.1073/pnas.2012002117

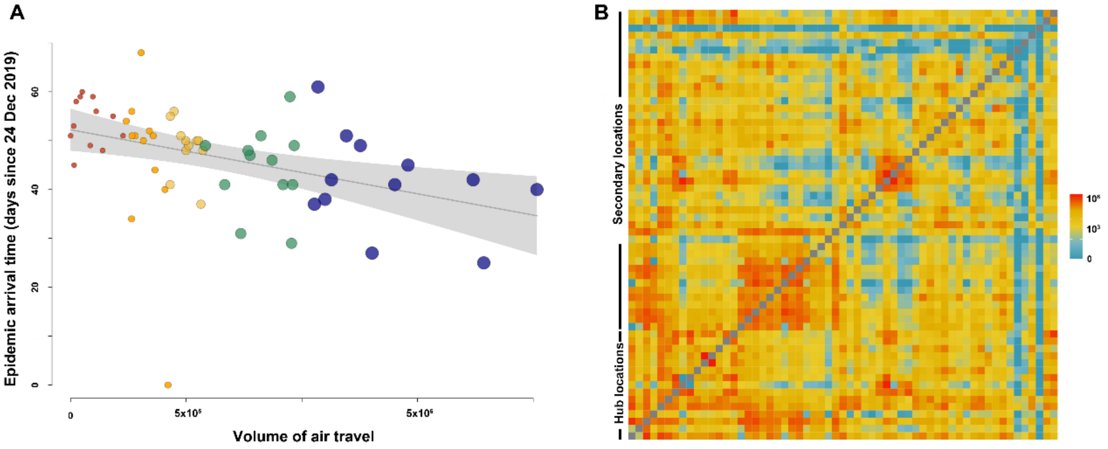

Fig. S1. Differences among locations in air travel. (A) Locations with larger volume of air travel tend to have earlier arrival of local transmission. The arrival time is projected as the days since the first arrival of epidemics in Wuhan, i.e. 24 December 2019. (B) Dynamics of air travel between all high-risk locations in 2019.

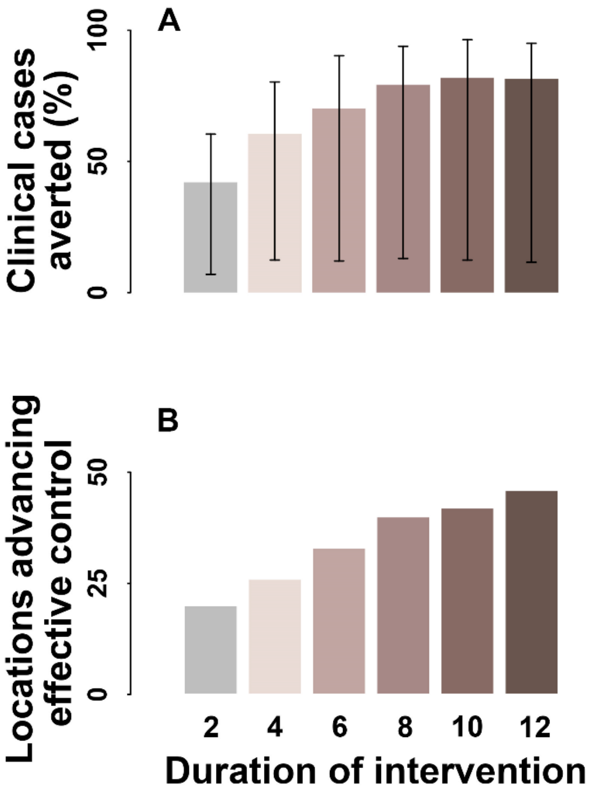

Fig. S2. Intervention effect through moderate intensity among secondary high-risk locations. Intensive intervention was implemented in the GIH, followed by moderate intervention among secondary locations. Effectiveness is evaluated by (A) the proportion of clinical cases averted and (B) the number of locations with accelerated achievement of effective control of transmission.

Table S1. Details of 59 high-risk locations analyzed in this study.

ID	Country	Location	Population	Climates*	Intervention Tier
1	USA	New York (NY)	8398748	NH-Temperate	Hub
2	USA	Bergen (NJ)	932202	NH-Temperate	Secondary
3	USA	Middlesex (MA)	1611699	NH-Temperate	Secondary
4	USA	Philadelphia (PA)	1584064	NH-Temperate	Hub
5	USA	Los Angeles (CA)	3990456	NH-Subtropical	Hub
6	USA	Cook (IL)	5150233	NH-Temperate	Secondary
7	USA	Wayne (MI)	1749343	NH-Temperate	Secondary
8	USA	Miami-Dade (FL)	2716940	NH-Subtropical	Secondary
9	USA	New Orleans (LA)	391006	NH-Subtropical	Secondary
10	USA	Fairfield (CT)	62105	NH-Temperate	Secondary
11	USA	Harris (TX)	4713325	NH-Subtropical	Secondary
12	USA	Fulton (GA)	1063937	NH-Subtropical	Secondary
13	USA	Prince George's (MD)	909327	NH-Temperate	Secondary
14	USA	Marion (OH)	36087	NH-Temperate	Secondary
15	USA	Washington DC	702455	NH-Temperate	Hub
16	USA	Marion (IN)	28047	NH-Temperate	Secondary
17	USA	Fairfax (VA)	24019	NH-Temperate	Secondary
18	USA	Denver (CO)	727211	NH-Temperate	Secondary
19	Spain	Community of Madrid	6661949	NH-Temperate	Secondary
20	Italy	Lombardy region	10088484	NH-Temperate	Secondary
21	Germany	Munich	1471508	NH-Temperate	Secondary
22	United Kingdom	London	8908081	NH-Temperate	Hub
23	France	Paris	2148271	NH-Temperate	Hub
24	Turkey	Ankara	5700000	NH-Temperate	Secondary
25	Iran	Tehran	16000378	NH-Temperate	Secondary
26	China	Wuhan	11081000	NH-Subtropical	Secondary
27	China	Shanghai	24281400	NH-Subtropical	Hub
28	China	Beijing	15563215	NH-Temperate	Secondary
29	China	Guangzhou	14904400	NH-Tropical	Secondary
30	China	Shenzhen	12905000	NH-Tropical	Hub
31	China	Wenzhou	3039439	NH-Subtropical	Secondary
32	China	Harbin	5115000	NH-Temperate	Secondary
33	China	Hongkong	7482500	NH-Tropical	Hub
34	Russia	Moscow	12506468	NH-Temperate	Secondary

35	Brazil	Sao Paulo	12176866	NH-Subtropical	Secondary
36	Canada	Montreal	1704694	NH-Temperate	Secondary
37	Belgium	Brussels	1208542	NH-Temperate	Secondary
38	Netherlands	North Brabant	2562566	NH-Temperate	Secondary
39	Switzerland	Zurich	415215	NH-Temperate	Hub
40	India	Mumbai	12478447	NH-Tropical	Hub
41	India	New Delhi	257803	NH-Subtropical	Hub
42	Portugal	Porto	287591	SH-Temperate	Secondary
43	Peru	Lima	8852000	NH-Tropical	Secondary
44	Ireland	Cork	210000	NH-Temperate	Secondary
45	Sweden	Stockholm	974073	NH-Temperate	Secondary
46	Austria	Vienna	1888776	NH-Temperate	Secondary
47	Israel	Jerusalem	919438	NH-Subtropical	Hub
48	Saudi Arabia	Riyadh	7676654	NH-Subtropical	Secondary
49	Japan	Tokyo	13929286	NH-Temperate	Hub
50	Singapore	Singapore	5638700	NH-Tropical	Hub
51	Chile	Santiago Metropolitan Region	7036792	SH-Subtropical	Secondary
52	Mexico	Mexico City	8918653	NH-Tropical	Secondary
53	Ecuador	Guayas	3645483	SH-Tropical	Secondary
54	Pakistan	Khyber Pakhtunkhwa	35525047	NH-Subtropical	Secondary
55	South Korea	Daegu	2489802	NH-Temperate	Secondary
56	Poland	Mazovian Voivodeship	5384617	NH-Temperate	Secondary
57	Romania	Suceava	92121	NH-Temperate	Secondary
58	Australia	Sydney Metropolitan Region	5312163	SH-Subtropical	Secondary
59	Malaysia	Selangor	6448400	NH-Tropical	Secondary

*NH: Northern hemisphere; SH: Southern hemisphere

Table S2. Best-fit model parameters for the simulation of seasonality of temperature across hemisphere and climates.

Hemisphere	Climates	Number of locations	Median estimate of parameters (95%CI)			
			a0	a1	b1	w
Southern	Temperate	1	6.522<br>(6.164, 6.881)	5.275<br>(4.51, 6.04)	3.738<br>(2.808, 4.668)	0.018<br>(0.017, 0.018)
Southern	Subtropical	2	16.85<br>(16.52, 17.19)	6.394<br>(5.928, 6.861)	1.859<br>(0.907, 2.811)	0.017<br>(0.017, 0.018)
Southern	Tropical	1	26.01<br>(25.95, 26.08)	-0.0664<br>(-0.237, 0.104)	1.173<br>(1.081, 1.265)	0.0200<br>(0.019, 0.021)
Northern	Temperate	34	11.37<br>(11.22, 11.53)	-10.93<br>(-11.17, -10.69)	-3.817<br>(-4.253, -3.381)	0.0172<br>(0.017, 0.017)
Northern	Subtropical	13	23.14<br>(21.81, 24.47)	-8.915<br>(-9.287, -8.543)	-1.427<br>(-2.341, -0.512)	0.0162<br>(0.016, 0.017)
Northern	Tropical	8	23.14<br>(21.81, 24.47)	-3.052<br>(-3.776, -2.329)	2.714<br>(0.195, 5.233)	0.0121<br>(0.009, 0.015)
